# A Subtle Alternative Splicing Event Gives Rise to a Widely Expressed Human RNase k Isoform

**DOI:** 10.1371/journal.pone.0096557

**Published:** 2014-05-05

**Authors:** Evangelos D. Karousis, Diamantis C. Sideris

**Affiliations:** University of Athens, Faculty of Biology, Department of Biochemistry and Molecular Biology, Athens, Greece; International Centre for Genetic Engineering and Biotechnology, Italy

## Abstract

Subtle alternative splicing leads to the formation of RNA variants lacking or including a small number of nucleotides. To date, the impact of subtle alternative splicing phenomena on protein biosynthesis has been studied in frame-preserving incidents. On the contrary, mRNA isoforms derived from frame-shifting events were poorly studied and generally characterized as non-coding. This work provides evidence for a frame-shifting subtle alternative splicing event which results in the production of a novel protein isoform. We applied a combined molecular approach for the cloning and expression analysis of a human RNase κ transcript (RNase κ-02) which lacks four consecutive bases compared to the previously isolated RNase κ isoform. RNase κ-02 mRNA is expressed in all human cell lines tested end encodes the synthesis of a 134-amino-acid protein by utilizing an alternative initiation codon. The expression of RNase κ-02 in the cytoplasm of human cells was verified by Western blot and immunofluorescence analysis using a specific polyclonal antibody developed on the basis of the amino-acid sequence difference between the two protein isoforms. The results presented here show that subtle changes during mRNA splicing can lead to the expression of significantly altered protein isoforms.

## Introduction

Alternative splicing appears to be the rule for higher eukaryotic genomes. It is estimated that approximately 95% of human multiple exon pre-mRNAs are differentially processed to yield multiple mRNA products [1], [2]. This fact underlines the dynamics of transcriptome plasticity, allowing for the evolutionary emergence of novel motifs and biological functions on the protein level by combining efficient structural features. Furthermore, in higher eukaryotes, several molecular mechanisms such as alternative splicing, alternative transcriptional initiation points, alternative polyadenylation and RNA editing compensate for the difference between the number of genes and expressed transcripts and proteins [3].

Alternative splicing events taking place at donor or acceptor sites located in close proximity to the conventional splice sites, give rise to mRNAs which differ by a few nucleotides [4]. The events leading to such subtle splice variants involve the utilization of alternative donor or acceptor splice sites, since polymorphisms on the corresponding splice sites eliminate these subtle alternative splicing events [5]. Concerning the molecular mechanisms involved, it has been proposed that the tandem pairs of alternative splice sites generally resemble normal splice sites. It appears that intrinsic properties of the spliceosome substances favor alternative splicing on tandem sites [6]. Subtle alternative splice sites bearing a NAGNAG motif (N standing for any nucleotide) have been shown to be the most frequent since approximately 2000 alternative spliced acceptors of this type have been observed in humans [7]. The most common distance length between two splice sites is 4 nucleotides long at the donor site. It has been suggested, though, that their frameshift impact gives rise to products that are predicted as non-sense mediated (NMD) targets (8).

It has been shown that alternative splicing occurring at NAGNAG sites generates important differences between the proteomes of mammalian tissues. This fact implies that the evolutionary paths of mammalian proteins are highly affected by the attribution of introns within the coding sequences of the genes. NAGNAG events observed in human tissues are often tightly regulated by sequence-specific determinants. Alternative splicing at tandem sites constitutes a subtle mechanism which allows the modification of protein products without affecting the stability of the existing transcripts. This fact seems to exert an accelerating force on protein evolution at exon-exon boundaries [8].

Recent experimental data have added a novel perspective in the transcriptome analysis by revealing an important group of transcripts termed long non coding RNAs (lncRNAs). lncRNAs are regulatory RNAs exceeding the length of 200 nucleotides [9]. Large-scale sequencing and prediction analyses of full length cDNA libraries have revealed that lncRNAs constitute an important portion of the total human transcriptome with an ever-rising number of reports reaching 23,000 transcripts [10]–[13]. Even though the mechanisms of lncRNAs biogenesis are quite diverse, their transcription and splicing are mediated similarly to protein-coding mRNAs, with the majority of them being 5′ capped and polyadenylated [14]. Concerning their function, lncRNAs seem to take part in transcriptional [15] and post-transcriptional regulation [16], epigenetic regulation by recruiting chromatin remodelling [17], whereas they seem to implicate in tumorigenesis mechanisms [18]–[22]. Given the relative abundance of lncRNAs and their features similarity with mRNAs a crucial issue considering novel poly(A)^+^ isolated mRNAs is whether they are protein coding or not.

Human RNase κ is a previously established protein belonging to a family conserved in all metazoans. The high conservation of all members along with the fact that the human enzyme is widely expressed in all tissues and developmental stages [23] suggest a very important biological function that is currently under investigation. Human RNase κ is represented by a single copy in the human genome and retains the pattern of gene organization that is preserved by almost all known members of the gene family, consisting of three exons interrupted by two introns [24]. The human representative exhibits *in vitro* endoribonucleolytic activity cleaving preferentially ApG and ApU phosphodiester bonds, while it hydrolyzes UpU bonds at a lower rate [23]. Reducing reagents and site-directed mutagenesis experiments showed that a disulfide bond between cysteine residues 6 and 69 is essential for the ribonucleolytic activity of the enzyme [25].

The present work reports a human subtle alternative splicing event giving rise to a protein coding mRNA variant (RNase κ-02) that lacks 4 nucleotides compared to the previously reported RNase κ mRNA isoform. Cloning and expression analysis of this subtly alternatively spliced mRNA was achieved by the development of a modified hybrid selection approach which may provide a tool for the study of similar cases. RNase κ-02 mRNA encodes the synthesis of an alternative protein isoform in human cell extracts which follows a cytoplasmic distribution.

## Results

### RNase κ expression in human cell lines

**Figure 1:**
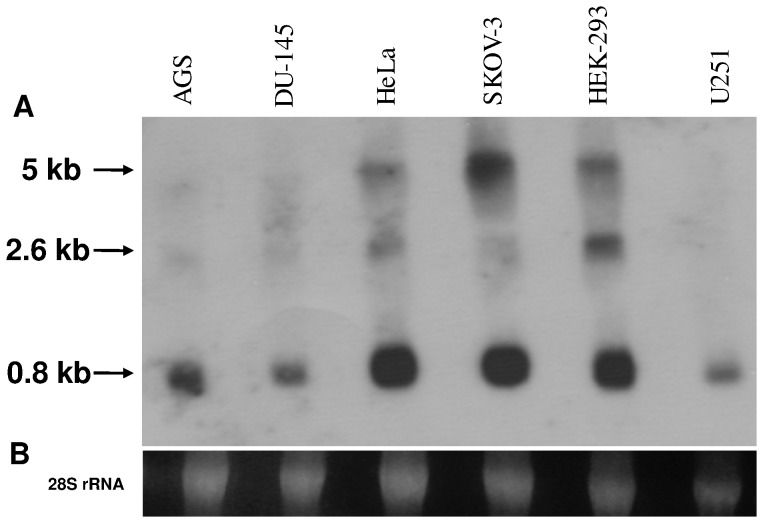
Northern blot analysis of RNase κ in human cell lines. Approximately 20 µg of total RNA isolated from 6 human cell lines were loaded per lane, separated by electrophoresis in a 1.2% agarose-formaldehyde gel and transferred to a Porablot NY plus Nylon membrane. The membrane was hybridized with a ^32^P-labelled cDNA probe corresponding to nucleotides 1–466 of the human RNase κ cDNA sequence (accession number AM746459.1). Sizes were estimated by comparison with RNA size markers. (B) 28S rRNA was used as a control for the amounts of RNA loaded.

In a previous work we reported the identification and isolation of a cDNA sequence of 466 bp (RNase κ, hereinafter designated as RNase κ-01) encoding the synthesis of a 98 amino-acid protein exhibiting endoribonucleolytic activity [23]. An EST and deep RNA sequencing data *in silico* analysis revealed that RNase κ-01 is expressed in a large variety of normal and pathological tissues in all developmental stages. In order to investigate the expression pattern of the human *RNase κ* gene, a Northern blot analysis was performed using total RNA isolated from various human cell lines (Figure 1). Our results show that a main band of approximately 0.8 kilobases is expressed at different levels in all cell lines examined, while two additional bands of approximately 2.6 and 5 kb appeared in some cases.

In addition, a TBLASTN search of the available EST sequence databases using the RNase κ-01 amino acid sequence as query resulted in the retrieval of a large number of EST sequences among which an important subset, distributed in a wide variety of tissues, differs from the RNase κ-01 cDNA only in the absence of 4 consecutive bases. It is conceivable that no information concerning the expression of this putative isoform could be deduced by Northern Blot analysis.

### RNase κ-02 mRNA isolation and cloning by means of a novel hybrid selection strategy

**Figure 2:**
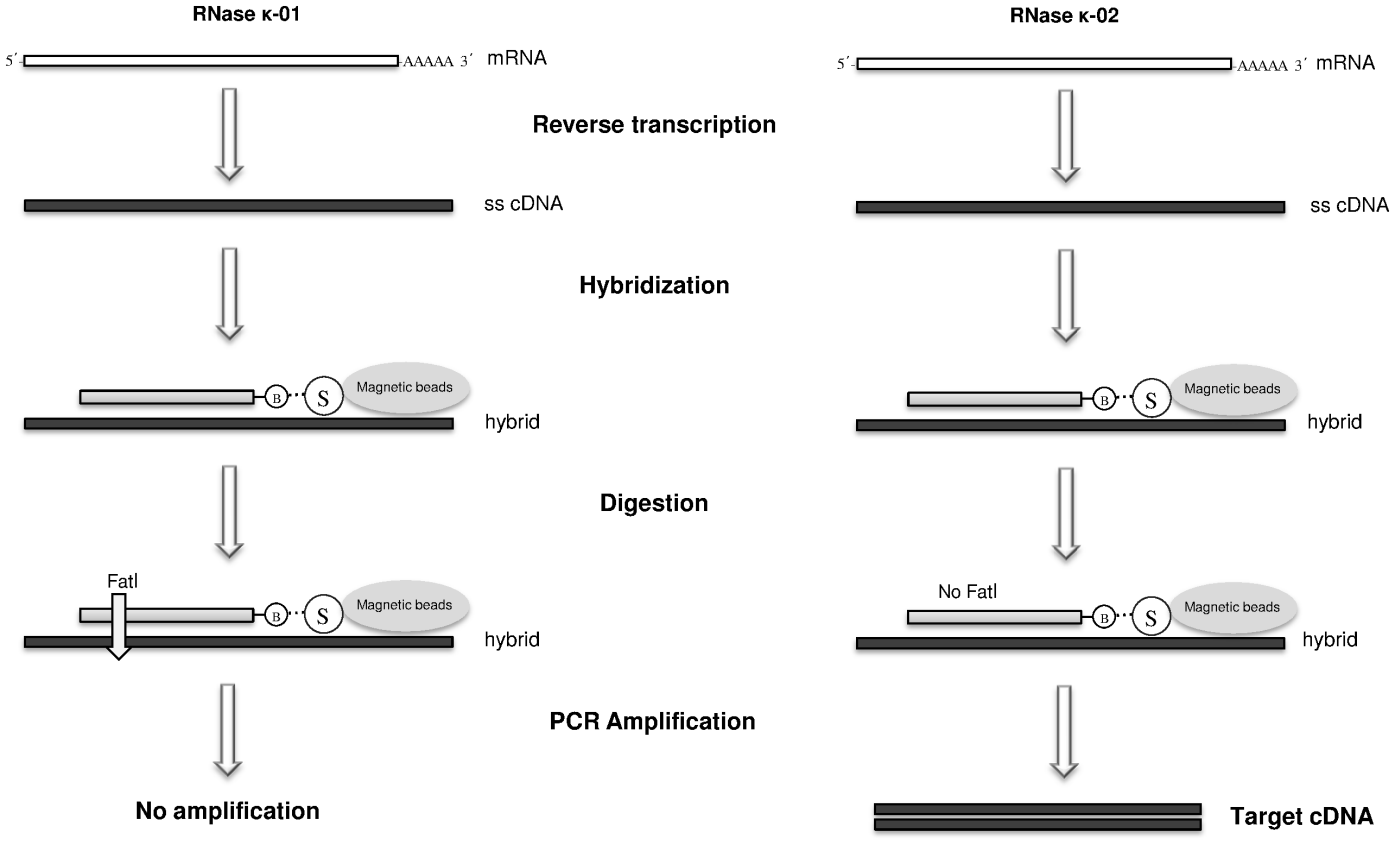
Schematic representation of the human RNase κ gene, its derived mRNA isoforms and amino acid sequence alignment of the protein isoforms. (A) White boxes represent gene exons and the horizontal bold lines introns. In mRNAs, the black parts of the rectangles demonstrate the protein-coding regions and the white the non-coding regions. The grey line represents the GTTG sequence that is present in RNase κ-01 isoform. The positions of ATG initiation codons are marked on both isoforms. (B) Amino acid sequences are numbered on the right and the identical portion of the two proteins is marked in grey frame.

Conventional RT-PCR experimental approaches do not facilitate the selective isolation between two very closely related mRNA variants such as RNase κ-01 and RNase κ-02 mRNA isoforms, which differ only in 4 nucleotides. For this reason, aiming at the isolation and cloning of the RNase κ-02 variant we devised and implemented a new hybrid selection methodology outlined in Fig. 2. This approach enables the isolation and cloning of subtle alternative transcripts on the basis of their sequence difference.

More specifically, a poly(A)^+^ cDNA library from human HEK-293 cells was prepared and hybridized with a 5′ biotinylated single stranded DNA probe corresponding to a portion of the RNase κ-01 cDNA coding region. Two types of hybrids are formed in the hybridization pool, hybrids which are fully complementary and hybrids that are partially complementary with the cDNA probe. Since the absence of GTTG sequence eliminates a FatI restriction site, only hybrids corresponding to RNase κ-02 isoform remain unaffected after the complete digestion with FatI. The selected hybrids were amplified by PCR and were cloned into the pCR 2.1 vector. Seven cDNA clones were selected end verified by restriction endonuclease mapping and sequencing analysis. Sequencing results (NCBI database, Acc. Number: KF980888) revealed no differences among these clones, which additionally appear to be identical to portions of two other mRNA sequences submitted in the NCBI database (Acc. Number BC070349.1 and BC095436.1).

**Figure 3:**
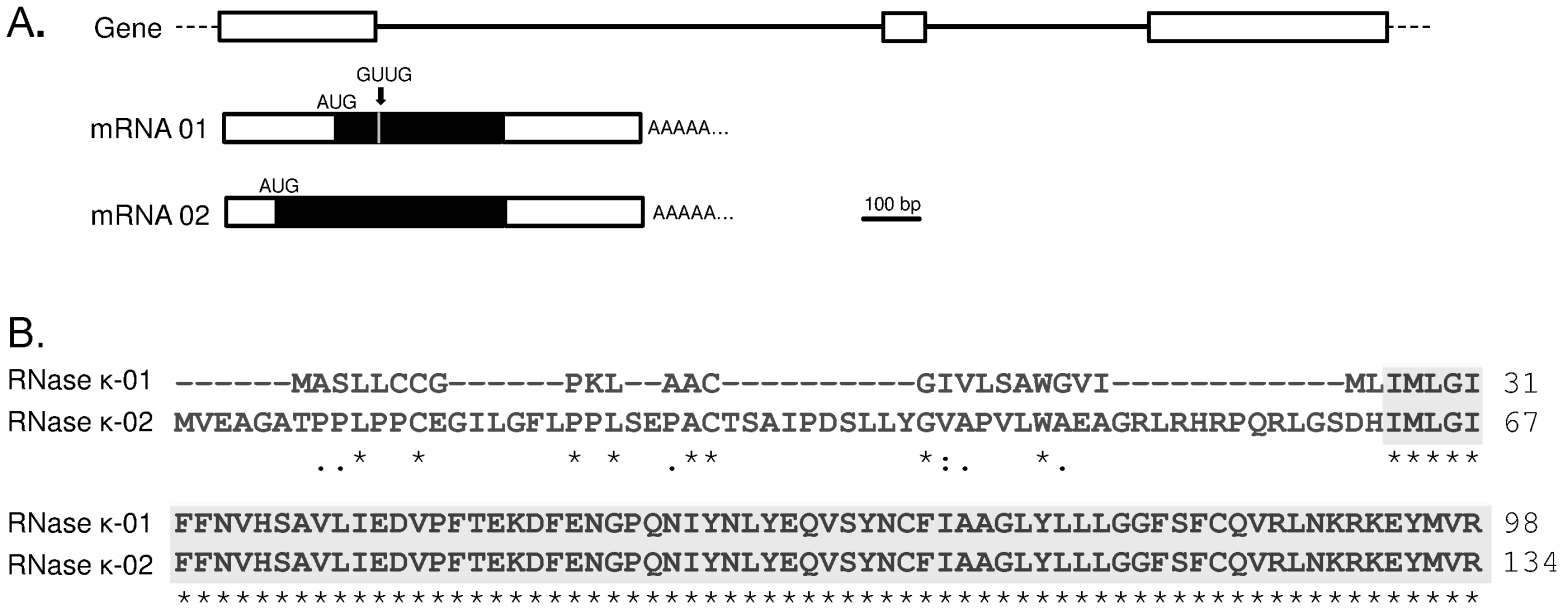
Schematic representation of the hybrid capture protocol for the selection of RNase κ-02 alternative transcript. The method consists of the following steps: (i) Reverse transcription of poly(A)^+^ mRNA and construction of a single stranded cDNA library. (ii) Hybridization of cDNA molecules with a biotinylated single stranded RNase κ specific DNA probe immobilized on streptavidin magnetic beads. (iii) Complete digestion of the hybrids with an appropriate restriction enzyme. (iv) PCR amplification of the selected target cDNA.

A subtle alternative splicing event occurring at the exons 1 and 2 junction site was revealed by alignment of the isolated clone with the human *RNASEK* gene sequence. As a result of this event, the sequence GTTG is absent in RNase κ-02 cDNA compared to RNase κ-01 as presented in Fig. 3A.

The isolated clone is 540 bp long and harbors an ORF of 405 nucleotides, encoding a 134-amino acid protein with a calculated mass of 14.900 Da. This protein occurs from the use of an upstream initiation codon compared to the previously reported κ-01 isoform. The probability of this codon encoding the initiator methionine is very high (68%) according to an initiation codon analysis performed using the ATGpr program [26]. Protein alignment of the deduced amino acid sequences of the two RNase κ isoforms revealed no similarities within their amino-terminal portion whereas the 63–134 amino acid region of RNase κ-02 is absolutely identical to the 27–98 amino acid part of RNase κ-01 (Fig. 3B).

### Relative quantitative expression analysis of RNase κ-01/RNase κ-02 mRNA isoforms

**Figure 4:**
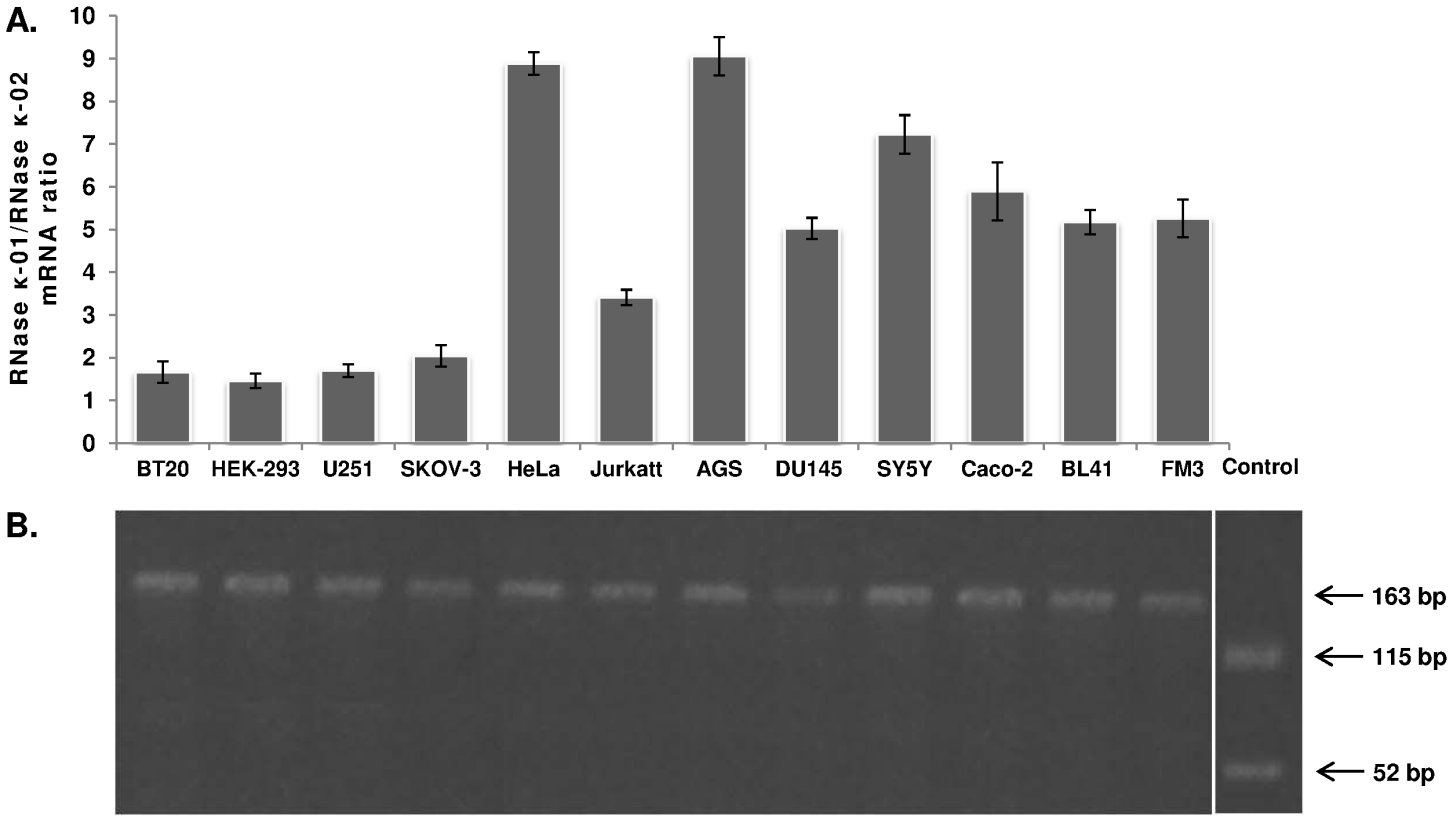
RNase κ-01/RNase κ-02 mRNA ratio in human cell lines. (A) A histogram of RNase κ mRNA isoforms ratio. Total RNA isolated from 12 human cell lines was reverse transcribed and amplified for 10 cycles by regular PCR. Equal amount of the PCR products from each reaction were incubated in the presence or in the absence of FatI and the samples were re-amplified by Real Time PCR. The comparative ΔC_T_ analysis performed as described in Materials and Methods resulted in the relative quantification of RNase κ mRNA isoforms. Error bars denote the standard error of the mean of triplicate reactions performed three times for each cell line. (B) The Real Time PCR products from the digested samples were overnight incubated with FatI and analyzed by electrophoresis in a 2% agarose gel. In a parallel experiment, 200 ng of RNAse κ-01 cDNA amplified by the same primers were digested under the same conditions as a control reaction.

Alternative splicing is a highly regulated molecular process and in some cases specific splice variants are expressed under varying conditions. Accordingly, the observed expression ratio between two short-distance tandem sites splice variants is affected by cell and tissue type, developmental stages and external stimuli [27]. To this end, the relative expression ratio of the RNase κ-01/RNase κ-02 mRNA isoforms was examined. Since the use of isoform-specific primers in a broad spectrum of RT-qPCR amplification conditions did not provide specific products, we applied an alternative molecular approach. More specifically, total RNA was reverse transcribed and amplified for 10 cycles by PCR using a set of primers on both sides of the exons 1–2 junction site. This step renders both isoforms double-stranded without affecting their initial ratio. Next, equal volumes of the PCR product are incubated overnight in the presence or in the absence of the FatI restriction enzyme. As mentioned above, the products corresponding to RNase κ-01 mRNA are digested, whereas PCR products corresponding to RNase κ-02 remain unaffected. On the other hand, in the absence of FatI both populations remain unaffected. Finally, these sample couples undergo Real-Time PCR amplification and their C_T_ differences reveal the RNase κ-01/RNase κ-02 mRNA ratio. Three independent mRNA preparations from 12 human cell lines were analyzed as mentioned, and the obtained results are represented in Fig. 4A.

Quantitative analysis of the two transcripts relative abundance portrayed the expression of the RNase κ-02 isoform in all cases, with a ratio of RNase κ-01/RNase κ-02 mRNA ranging from 1.45 (HEK-293 cells) to 9.06 (AGS cells).

For the accurate interpretation of the quantification data it is important to verify that only RNase κ-02 cDNA molecules were amplified after the digestion step. For this reason, the Real Time PCR products underwent an additional digestion with FatI and were analyzed by agarose gel electrophoresis. As demonstrated in Fig. 4B, a unique product of the expected size was detected in all cases, denoting the reliability of our results.

### RNase κ-02 protein is expressed in human cell lines

It is well established that an important portion of the transcriptome has no protein-coding capacity [28]. Therefore, a significant question raised was whether RNase κ-02 transcript is protein coding or not.

Given that the RNase κ-02 amino-terminal portion (1-62 aa) is different to the RNase κ-01 protein and bears no important similarities with other known human proteins, we proceeded to the production of a specific polyclonal antibody that recognizes only the RNase κ-02 protein isoform. For this reason, the amino-terminal portion of the RNase κ-02 was expressed as a fusion protein with intein in *E.coli* expression system as described in Materials and Methods. After cleavage of the fusion protein with β-mercaptoethanol, the desired peptide was isolated and used for the immunization of two rabbits. The serum was affinity – purified against the target peptide and the purified IgGs (K02N antibody) were collected. When a series of total cell extracts (HEK-293, AGS, HeLa, SH-SY5Y and FM3 cell lines) underwent Western Blot analysis, no bands were detected. Moreover, no immunodetection signals were observed even when a commercially available antibody against the common carboxy-terminal region of RNase κ-01 and RNase κ-02 protein isoforms were used (data not shown).

**Figure 5:**
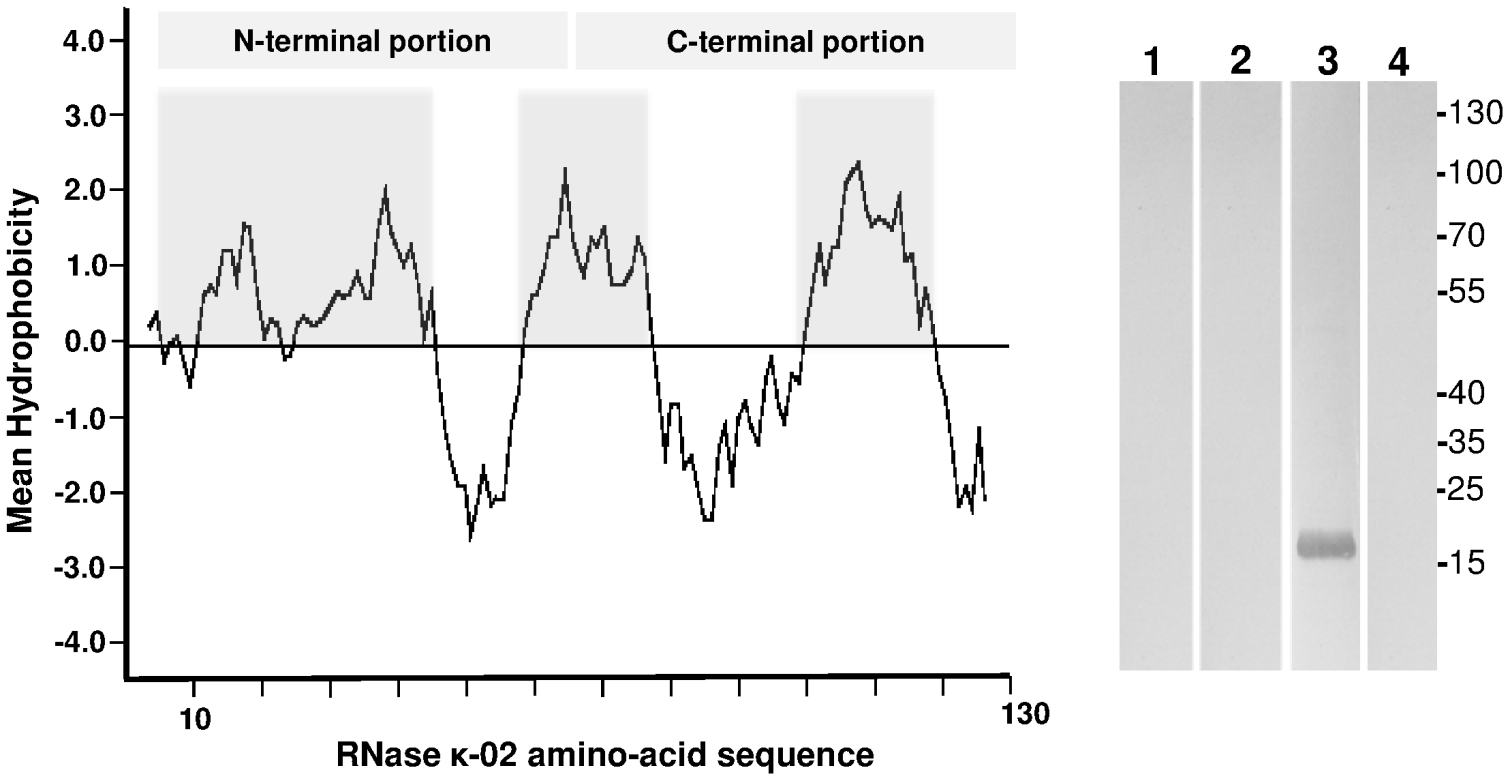
RNase κ-02 hydrophobicity plot and Western blot analysis of HEK-293 protein fractions. (A) Kyte-Doolittle Hydrophobicity plot of the RNase κ-02 protein. Regions with values above 0 are hydrophobic in character and are marked in grey boxes. (B) Western blot analysis of normal and RNAi depleted RNase κ-02 HEK-293 protein fractions collected by Triton X-114 phase separation. Twenty micrograms of proteins from detergent-depleted phase (lane 1), detergent-enriched phase (lane 2) and insoluble fraction (lane 3) from normal cells and insoluble fraction of RNAi depleted RNase κ-02 cells were analyzed by SDS/PAGE, electro-transferred onto a nitrocellulose membrane. RNase κ-02 was detected using the specific K02N polyclonal antibody. A set of marker proteins of known molecular weight were run in parallel.

RNase κ-02 is highly hydrophobic according to protein prediction analysis. As shown in the Kyte-Doolittle hydrophobicity plot [29], RNase κ-02 protein bears 3 highly hydrophobic regions (Fig. 5A). The first region is located within the amino-terminal portion that differs from RNase κ-01, whereas the other two hydrophobic areas lie within the common carboxy-terminal region of the two isoforms.

Based on RNase κ-02 relative hydrophobicity, we opted for a strategy that would allow us the considerable enrichment of highly hydrophobic proteins in cell extracts. To achieve this goal, we proceeded to the temperature-induced phase separation using Triton X-114, which is widely employed in similar cases [30], [31]. This process separates proteins according to their relative hydrophobicity leading to the separation of hydrophilic molecules in the detergent-depleted (aqueous) phase, amphipathic integral membrane proteins are recovered in the detergent-enriched phase and insoluble proteins are pelleted after centrifugation. Treatment of HEK-293 cell homogenate with Triton X-114 and phase separation was followed by SDS-PAGE analysis and immunodetection using the K02N polyclonal antibody of the three protein fractions. As shown in figure 5B, the analysis resulted in the recovery of RNase κ-02 protein in the detergent-insoluble fraction (lane 3), whereas no bands were observed in the aqueous (lane 1) or in the detergent-enriched (lane 2) fractions. In a parallel experiment, no signal was detected in the detergent insoluble franction prepared from RNase κ-02 RNAi knock-down HEK-293 cells (lane 4), a fact denoting the specificity of the utilized antibody against RNase κ-02. In all cases, control experiments were performed adding only secondary antibody and no bands were observed (data not shown). RNase κ-02 protein exhibited a molecular mass of ∼16 kDa which is in accordance with the calculated mass deduced from its amino-acid sequence (14,900 Da).

**Figure 6:**
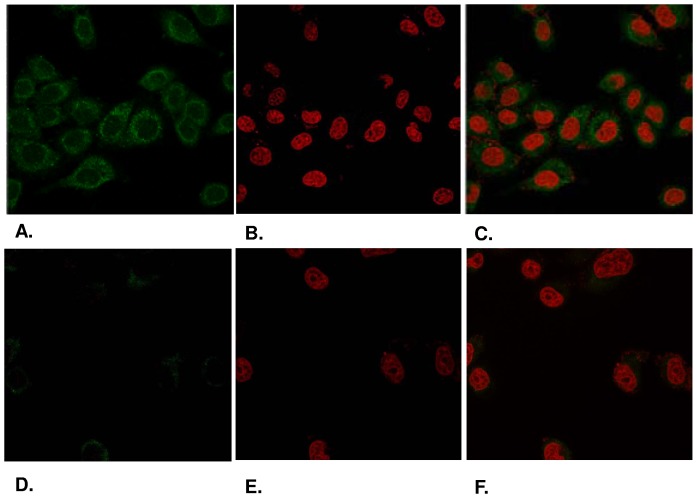
Immunofluorescence microscopy analysis of RNase κ-02. HEK-293 cells (A) and RNAi depleted RNase κ-02 HEK-293 cells (B) were treated with K02N specific polyclonal antibody and protein – primary antibody complexes were visualized with rabbit matched Alexa-488 secondary antibody. DNA was stained with propidium iodide (B,E). Merged image of the two stains (C,F).

Finally, in order to analyze the subcellular distribution of the RNase κ-02 protein we proceeded to an immunofluorescence analysis using the Κ02Ν polyclonal specific antibody. As shown in figure 6, RNase κ-02 seems to be distributed only in the cytoplasm (Fig. 6A), since its fluorescence signal does not overlap with the iodium propide signal used to stain the cell nucleus (Fig. 6B). In order to verify this finding, we performed RNAi mediated knock down of human RNase κ-02. HEK-293 cells that were transfected with shRNA-expressing plasmids (iK3) and were subsequently fixed, permeabilized and incubated with the K02N antibody under the same conditions. As shown in Fig. 6D, after the knock down of RNase κ-02 the immunofluorescence signal was virtually depleted, a fact that validates the cytoplasmic distribution of RNase κ-02 protein isoform.

## Discussion

Subtle alternative splicing events are being observed in an ever-rising number of human genes, as depicted by the exponential accumulation of EST and high throughput RNA sequencing data [6], [32]–[34]. The impact of subtle alternative splicing on protein products depends on whether they are frame-shifting (Δ2, 4, 5, 7, 8, etc.) or frame-preserving (Δ3, 6, 9, etc.) with Δ denoting the sequence length between the tandem splice sites. Frame-preserving tandem sites result in the addition or absence of only a few amino-acids, leading to the production of similar protein isoforms which may bear different functional properties. For instance, a Δ3 subtle alternative splicing event occurring in *ATN1* human gene product determines the cellular topology (nuclear or cytoplasmic) of the corresponding protein isoforms [27]. At the donor site, alternative splicing events in which the distance between two splice sites is 4 nucleotides long are the most frequent. However, their frameshift effect often creates mRNA isoforms that are predicted as non-sense mediated decay (NMD) targets [4] and therefore these cases have not been systematically investigated.

The methodological approach presented allowed us to achieve the cloning of a human RNase κ mRNA variant that occurs after a Δ4 splicing event. This experimental strategy offers several advantages for the study of transcripts generated by alternative splicing compared to conventional RT-PCR approaches. The key point of our procedure is the addition of a digestion step with an appropriate restriction enzyme which specifically acts against complementary sequences, allowing for the selective amplification of the unaffected molecules. Moreover, the application of a hybrid selection step permits the isolation of rare transcripts in terms of sequence specificity. Abundant RNA molecules such as ribosomal RNAs and highly expressed mRNAs that may hinder the selective isolation of low expression transcripts are removed during the washing steps and the amplification of target molecules is facilitated. Based on the above, this protocol may prove valuable for the experimental validation of a broad spectrum of transcripts by selecting a digestion enzyme that recognizes the non-desired target. Along these lines it is conceivable that the elimination of a unique restriction site could provide the selection edge required for the isolation of a desired alternative transcript.

Sequence alignment of the isolated RNase κ-02 cDNA with the human *RNASEK* gene revealed that the RNase κ-02 mRNA isoform occurs as a result of an alternative Δ4 donor event within the first intron, a phenomenon which is considered to be quite frequent in human gene products [4], [35].

It has been proposed that differential regulation of subtle alternative splicing isoforms expression levels may denote function. Particularly, the ratio between the two short-distance splice isoforms may differ in various tissues and cell types [34], [36], depending on developmental stages [37], [38] or in response to external factors [39]. To date, a variety of methodological implementations such as polyacrylamide [40], [41] and agarose [27], [42] gel electrophoresis, capillary electrophoresis [43] as well as RNA sequencing analysis [8] have been employed in order to specify the expression ratio. However, these approaches cannot provide accurate results or they are limiting due to the sophisticated equipment requirements.

The Real-time PCR based methodological approach presented here may be applied for the expression analysis of variant sequences harboring distinctive restriction sites. In other words quantification, similarly to cloning is achieved in terms of the isoforms sequence differentiation. The results of our analysis demonstrated that RNase κ-02 mRNA is expressed in all the examined cell lines with varying degrees of isoforms expression ratio. This observation may reflect the existence of differential regulation mechanisms that are implicated in the expression pattern of the human *RNASEK* gene.

It is well known that apart from protein coding RNAs, an important percentage of transcription products bears no protein coding capacity [28]. Apart from the widely studied non coding RNA populations such as rRNAs, tRNAs and microRNAs, long non coding RNAs (lncRNAs) consist a novel group of RNAs sharing a single common feature: a size of over 200 nucleotides [44]. A large number of lncRNAs bear mRNA signatures such as 5′cap and poly(A) tail [45] that seem to participate in their turnover [46]. For this reason, a crucial issue in this study was to assess whether RNase κ-02 mRNA isoform is protein coding or not.

The isolated RNase κ-02 cDNA clone contains an ORF of 405 nucleotides and encodes the synthesis of a 134-amino acid protein by utilizing an alternative initiation codon. It should be noted that according to a recently published study [47], there are at least 1849 human transcripts that can be translated by alternate ATG initiation codons. Its 1-62 amino-terminal portion bears no similarity with the previously characterized RNase κ-01 isoform or other human proteins, whereas the 63–134 region is absolutely identical to the major part of RNase κ-01 (aa 27-98). Based on this amino-acid sequence, we managed to produce and purify an RNase κ-02 specific polyclonal antibody. By the means of our newly developed antibody we were able to demonstrate the biogenesis of the RNase κ-02 protein resulting from the RNase κ-02 mRNA translation in human cells. This alternative protein isoform bears a cytoplasmic topology. To our knowledge, this is the first instance of a human protein isoform encoded by a Δ4 subtly alternatively spliced transcript.

The finding that RNase κ-02 protein isoform is detected only in the detergent-insoluble fraction of cell extracts after Triton X-114 phase separation is in agreement with the predicted highly hydrophobic nature of this protein. This fact, in combination with its cytoplasmic localization, supports the hypothesis that RNase κ-02 could participate in the formation of macromolecular complexes *in vivo* or localize in membranic structures such as the endoplasmic reticulum.

The work presented here highlights the dynamic plasticity of the existing transcriptome by the observation that a subtle change at the mRNA level can manifest as a profound change in protein content. The finding that a Δ4 alternative splicing event gives rise to a protein coding isoform challenges the notion that other similar products constitute NMD targets (8). Thus, these products may evade NMD by utilizing an alternative initiation codon or may constitute long non-coding RNAs with unattributed functions. Further investigation of frame-shifting subtle alternatively spliced variants in human or other organisms' transcriptomes may shed light to the functional role of these molecules.

## Materials and Methods

### Materials

Oligonucleotide synthesis and sequence analysis were performed by VBC-Genomics (Vienna, Austria). Restriction enzymes, IMPACT-CN expression system and chitin column were purchased from New England Biolabs (Hitchin, UK). Plasmid preparation kits and porablot NY plus Nylon membrane were from Macherey-Nagel (Düren, Germany). pCR 2.1 cloning vector was purchased from Invitrogen (Carlsbad, CA, USA). M-MLV reverse transcriptase was obtained from Clontech Laboratories (Palo Alto, CA, USA) and AMV RT XL reverse transcriptase was from TaKaRa (Tokyo, Japan). [α-^32^P] ATP was obtained from Izotop Ltd (Obninsk, Russia). Dynabeads oligo(dT)_25_ and Dynabeads M-280 streptavidin were from Dynal (Oslo, Norway). Alkaline phosphatase conjugated goat anti-rabbit IgG were purchased from Sigma-Aldrich (St Louis, MO, USA). Goat anti-rabbit IgG (H+L) Alexa Fluor 488 was obtained from Life technologies (Carlsbad, California, USA). RNase K Antibody (E-13) was obtained from Santa Cruz Biotechnology, Inc. (Dallas, Texas, USA). Marker proteins for SDS/PAGE molecular weight estimation were obtained from Fermentas Life Sciences (Vilnius, Lithuania). All other reagents used were of analytical grade and were purchased from Merck (Darmstadt, Germany).

### Cell culture, stable transfections and RNAi

In the present study the following cell lines were used: BT-20 (human breast adenocarcinoma cell line), AGS (gastric adenocarcinoma), DU145 (prostatic carcinoma), HeLa (cervical carcinoma), SKOV-3 (ovarian carcinoma), HEK-293 (embryonic kidney) and U251 (glioma), Jurkatt (immortalized T lymphocytes), SH-SY5Y (neuroblastoma), Caco-2 (epithelial colorectal adenocarcinoma), BL41 (Burkitt lymphoma) and FM3 (melanoma) under optimal growth conditions according to ATCC (American Type Culture Collection) guidelines. All the above commercially available human cell lines were obtained from ATCC. The plasmid iK3-pRS (HuSH™, OriGene) encodes the synthesis of an shRNA targeting the human RNase κ sequence 5′-GAGCAAGTCAGCTACAACTGTTTCATCGC-3′. As a control, a plasmid lacking the shRNA sequence was applied (pRS). 2×105 HEK-293 cells were seeded into six well plates and transfected with 5 µg pRS or iK3 plasmid with Xfect Transfection Reagent (Clontech, CA, USA). For the selection of stably transfected cell clones cells were grown in full medium containing puromycin (0.5 µg/mL) for 21 days. The efficiency of the knockdown was assessed on the mRNA level by quantitative RT-PCR (data not shown).

### Northern blot analysis

Total RNA (20 µg) extracted from various human cell lines using Trizol LS (Invitrogen) reagent were resolved by electrophoresis in 1.25% agarose gel containing 1.25 M phormaldehyde and transferred to a porablot NY plus Nylon membrane. The membrane was hybridized with a ^32^P-labelled probe corresponding to the previously isolated human RNase κ cDNA (accession number AM746459.1) at 42°C in ULTRAhyb buffer (Life Technologies, Carlsbad, California, U.S.). After hybridization, the membranes were washed and exposed to X-ray film at −80°C for 1 day with two intensifying screens.

### Identification of RNase κ-02 ESTs

The dbEST (Expression Sequence Tags) database [48] on the National Center for Biotechnology Information World Wide Web server was searched by the TBLASTN application. Multiple sequence alignment was performed by the ClustalW2 program [49] using default parameters.

### Isolation of the RNase κ-02 cDNA clone

Total RNA was prepared from cells and poly(A)^+^ RNA was isolated using Dynabeads oligo(dT)_25_. Poly(A)^+^ RNA (1 µg) was reversed transcribed by AMV RT XL using oligo(dT)_12–18_ as primers at 45°C for 40 min, followed by an additional incubation at 55°C for 20 min. In order to isolate the RNase κ-02 cDNA, a modified version of a hybrid selection technique developed in our laboratory [50] was employed. A 5′-biotinylated DNA probe complementary to the nucleotides 1–287 portion of the previously submitted RNase κ cDNA (accession number AM746459.1) was synthesized by PCR. The biotinylated strand of the PCR product was attached to Dynabeads M-280 streptavidin and hybridized overnight to the single-stranded cDNAs at 42°C in ULTRAhyb buffer (Life Technologies). Following the hybridization, the beads were selected and washed twice in 2× SSC, 0.1% SDS, twice in 0.2× SSC, 0.1% SDS for 5 min each at 42°C and once in TE buffer (10 mM Tris-HCl, pH 7.4, 1 mM EDTA) with 0.1% Tween-20 at room temperature. The collected beads were washed against 1× NEB2 buffer (New England Biolabs), resuspended in 20 µL of the same buffer containing 3 Units of FatI and incubated at 55°C overnight under constant agitation. The selected hybrids were amplified by PCR using RNase κ – specific oligonucleotides NdeFAlt (5′–ATG GTT GAG GCC GGG GCC A–3′) and RH (5′-GAA GGG ATT CAG TCT CTC GC-3′). The PCR products were cloned into the pCR 2.1 cloning vector and the isolated clones were sequenced in both directions.

### Relative quantitative expression analysis of RNase κ-01/RNase κ-02 mRNA isoforms

Two micrograms of total RNA isolated from a series of human cell lines were reverse transcribed by M-MLV RT using an RNase κ specific reverse primer (RH: 5′-GAA GGG ATT CAG TCT CTC GC-3′). The reaction products were amplified for 10 cycles in a 50 µl PCR reaction using the gene specific primers HumC1F (5′-GCG TCG CTC CTG ΑGC TGT GGG CCG AAG-3′) and HumC5R (5′-GAG CCG AAC TTG GCT GAA AGA GAA GCC TCC-3′). Two aliquots (15 µl each) of the PCR products were incubated overnight at 55°C with or without 1.5 Units of *Fat*I in a 20 µl reaction.

One milliliter of each of the above products was amplified by Real Time PCR using SYBR Green Chemistry and a set of internal RNase κ – specific primers (HumC3F: 5′-GAA GCT GGC CGC CAG CGG CAT CGT-3′ and HumC4R 5′-GCC TGC AGC GAT GAA AGA GTT GTA GCT GAC TTG C-3′). The reactions were performed in an ABI PRISM 7500 sequence detection system (Applied Biosystems) including a denaturation step at 95°C for 5 min followed by 40 cycles of 95°C for 15 sec and 60°C for 1 min. A final step was carried out for the production of a dissociation curve to ensure that the desired amplicon was detected and to confirm the absence of nonspecific products and/or primer dimers. Each reaction was performed in triplicate to evaluate data reproducibility and the whole procedure was repeated three times for each cell line.

The RNase κ-01/RNase κ-02 transcripts ratio into the amplified by regular PCR reaction cDNA pool was calculated by using the comparative ΔC_T_ method. In this method, 2^−ΔCT^ expresses the fold change in the expression of the target cDNAs between digested and non-digested samples. In digested samples, the C_T_ values correspond to the number of copies of RNase κ-02 cDNA whereas the C_T_ value in non-digested samples derives from both RNase κ populations. It should be noted that through Real-Time PCR control experiments using the above primer sets, the two cDNA isoforms are equally amplified and the linearity between the cDNA quantity and the C_T_ is validated for concentrations 10 to 10^8^ copies/µl.

### RNase κ-02 specific antibody production

A polyclonal antibody was raised against the 1–62 amino-terminal portion of the RNase κ-02 protein. To this end, this portion was expressed as a heterologous fusion protein with intein using the IMPACT-CN expression system, which utilizes an inducible self-cleavage activity to release the target protein from the affinity tag. The cDNA corresponding to the amino-terminal portion of RNase κ-02 was amplified using NdeI and XhoI-end specific primers (ΝdeFAlt: 5′– ATG GTT GAG GCC GGG GCC A – 3′, ΧhoR: 5′- CTC GAC GCG CAC CAT GTA TTC CTT G-3′). The PCR products were ligated to the similarly digested pTYB1 vector, yielding the ExK-02N construct. *E. coli* ER2566 host cells transformed with ExK-02N expression vector were inoculated into Luria-Bertani (LB) medium supplemented with 50 mg/l ampicillin and incubated at 30°C with shaking (250 rpm). When OD_600_ of the culture reached 0.5, IPTG was added to 0.3 mM final concentration and bacteria were harvested after a 6-hour induction period at 18°C. Cell pellets from 1 lit culture were resuspended in 20 ml of buffer A (20 mM Tris-HCl pH 8.0, 500 mM NaCl) and sonicated (6×1 min cycle). The lysate was then centrifuged at 15,000 *g* for 15 min at 4°C. The fusion protein was purified by affinity chromatography through its chitin binding domain. The clarified extract was loaded onto a chitin column (1 ml) pre-equilibrated with buffer A and then washed with the same buffer. After quickly flushing the column with 3 bed volumes of buffer A containing 50 mM β-merkaptoethanol (cleavage buffer), the flow was stopped and the column remained at 4°C for 40 hours. The target peptide of RNase κ-02 was eluted by adding 2 bed volumes of cleavage buffer and the fractions containing the larger amount of the target peptide were pooled and used for the immunization of two rabbits. The polyclonal antibody (K02N) production was performed by Pacific Immunology and the collected serum was affinity purified against the target peptide.

### Triton X-114 phase separation

Phase partition was performed according to the method of Bordier [51]. Briefly, HEK-293 cells were lysed in Triton X-114 cell lysis buffer (50 mM Tris pH 8.5, 150 mM NaCl, and 2% precondensed Triton X-114 containing protease inhibitors) and the suspension was incubated on ice for 30 min with frequent vortexing. The solution was centrifuged at 10,000 g at 4°C for 15 min to sediment the pellet fraction (insoluble fraction). The supernatant was collected, and incubated for 30 min at 37°C to achieve phase partitioning. After centrifugation of the mixture at 5,000 g at 25°C for 15 min, the upper detergent-depleted (aqueous) phase and the lower detergent-enriched phase were carefully collected. The protein concentration in each of the three fractions was estimated according to Bradford [52], using bovine serum albumin as standard.

### SDS/PAGE and Western blot analysis

Protein samples from Triton X-114 phase separation were analyzed by SDS /PAGE according to Laemmli [53] in 15% SDS polyacrylamide gels. For immunodetection, proteins were blotted onto a nitrocellulose membrane [54], which was subsequently incubated with K02N affinity purified specific polyclonal antibody. Primary antibody–antigen complexes were detected using goat anti-rabbit IgG conjugated to alkaline phosphatase and developed by 5-bromo-4-chloro-indolyl phosphate / nitroblue tetrazolium. In a parallel control expreriment, the blotted membrane was not incubated with a primary antibody.

### Immunocytochemistry and fluorescence imaging

HEK-293 cells were grown to 50% confluence on glass coverslips under optimum growth conditions as proposed by ATCC. The cells were fixed in PBS containing 4% formaldehyde for 15 minutes in room temperature, washed with PBS and permeabilized with PBS buffer containing 0.25% Triton X-100 for 10 minutes at room temperature. After washing with PBS, cells were blocked in PBS containing 0.1% Tween 20 and 1% BSA at room temperature and immunostained with K02N antibody for 1 hour at room temperature. Protein reacting with antibody was visualized with rabbit matched Alexa-488 secondary antibody. Cells were then incubated with RNase A for 20 minutes at 37°C, washed with PBS and incubated with propidium iodide at final concentration 1 µg/ml in PBS buffer for 4 minutes at room temperature to visualize nuclei. Fluorescence images of Alexa 488 were recorded with a confocal microscope (Confocal TE2000S/ECLIPSE C-1, Nikon, Tokyo, Japan) and appropriate filters for Alexa 488 with a 60× oil immersion objective lens. In parallel control experiments cells were not incubated with the primary antibody.
